# Performance evaluation of the FAST™ System and the FAST-PBC Prep™ cartridges for speeded-up positive blood culture testing

**DOI:** 10.3389/fmicb.2022.982650

**Published:** 2022-09-15

**Authors:** Alexia Verroken, Chaima Hajji, Florian Bressant, Jonathan Couvreur, Ahalieyah Anantharajah, Hector Rodriguez-Villalobos

**Affiliations:** Department of Microbiology, Cliniques Universitaires Saint-Luc, Université Catholique de Louvain, Brussels, Belgium

**Keywords:** Qvella, FAST™ System, positive blood cultures, direct MALDI-TOF MS, bacteremia, direct antimicrobial susceptibility testing, rapid resistance detection testing

## Abstract

**Objectives:**

As time to appropriate antimicrobial therapy is major to reduce sepsis mortality, there is great interest in the development of tools for direct identification (ID) and antimicrobial susceptibility testing (AST) of positive blood cultures (PBC). Very recently, the FAST™ System (Qvella) has been developed to isolate and concentrate microorganisms directly from PBCs, resulting in the recovery of a Liquid Colony™ (LC) within 30 min. The LC can be used as equivalent of an overnight subcultured colony for downstream testing. We aimed to evaluate the performances of the FAST™ System and FAST-PBC Prep™ cartridges by testing the resulting LC for direct ID, AST and rapid resistance detection.

**Materials and methods:**

Prospectively, FAST™ System testing was carried out on each patient’s first PBC with a monomicrobial Gram-stain result. In the second arm of the study, FAST™ System testing was carried out on blood cultures spiked with multidrug-resistant bacteria. Downstream testing using the LC included MALDI-TOF MS ID with the Bruker Biotyper^®^ smart system, rapid resistance detection testing including the Abbott Diagnostics Clearview™ PBP2a SA Culture Colony Test (PBP2a) and the Bio-Rad βLACTA™ Test (βLT). AST was performed using the Becton Dickinson Phoenix™ System or by Bio-Rad disk diffusion using filter paper disk following EUCAST 2020 breakpoint criteria.

**Results:**

FAST™ System testing was completed on 198 prospective PBCs and 80 spiked blood cultures. After exclusion of polymicrobial blood cultures, performance evaluation compared with standard of care results was carried out on 266 PBCs. Concordant, erroneous and no ID results included 238/266 (89.5%), 1/266 (0.4%), 27/266 (10.2%) PBCs, respectively. Sensitivity and specificity for PBP2a were 100% (10/10) and 75% (15/20), respectively. Sensitivity and specificity for βLT were 95.8% (23/24) and 100% (42/42), respectively. Categorical agreement for all 160 tested strains was 98% (2299/2346) with 1.2% (8/657) very major errors and 0.7% (10/1347) major errors.

**Conclusion:**

FAST™ System testing is a reliable approach for direct downstream testing of PBCs including MALDI-TOF MS ID, BD Phoenix™ and Bio-Rad disk diffusion AST as well as rapid resistance testing assays. Next steps include optimal integration of the FAST™ System in the PBC workflow with a view toward clinical studies.

## Introduction

Sepsis remains a worldwide cause of morbidity and mortality with a reported 49 million cases and an approximately 11 million avoidable deaths per year (World Health Organization, 2020). As time to appropriate antimicrobial therapy is a major factor to reduce sepsis mortality, there is a great interest in the development of tools for rapid identification (ID) and antimicrobial susceptibility testing (AST) of positive blood cultures (PBCs).

Direct ID from a PBC bottle is commonly applied in clinical microbiology laboratories either by matrix-assisted laser desorption ionization time-of-flight mass spectrometry (MALDI-TOF MS) or by molecular techniques. These approaches result in satisfactory analytical performances with a time saving of more than 24 h compared with overnight subculture ID results (Payne et al., 2018; Ruiz-Aragón et al., 2018). However, in this current era of increasing multi-drug resistance, ID results are frequently insufficient to decide on an optimal antimicrobial treatment and the use of rapid AST approaches remains more than ever essential. Manual methods, including cleaning, washing and concentrating microorganisms directly from the PBC to obtain a pellet for direct AST, have been used historically and studies have shown an overall categorical agreement above 90% providing results one day earlier compared to AST from subculture (Maelegheer and Nulens, 2017; Hogan et al., 2019; Infante et al., 2021). Furthermore, several commercial rapid AST systems relying on cellular imaging or turbidity measurements at consecutive points of time, have been developed and provide AST results for defined antibiotic within 5–7 h. Overall agreement with standard of care (SOC) AST has been reported between 88 and 98.7% for Gram-positive cocci and between 89.5 and 94.2% for Gram-negative bacilli (Charnot-Katsikas et al., 2017; Boland et al., 2019; Grohs et al., 2021).

Very recently, a novel approach has been developed called the FAST™ System (Qvella, Richmond Hill, Canada) designed to isolate and concentrate microorganisms directly from a PBC bottle, resulting in the recovery of a liquid colony (LC) within 30 min. Ultimately, the LC can be used as an equivalent of a solid subcultured colony, enabling use of downstream ID and AST systems available in the local clinical microbiology laboratory today. The objective of this study was to evaluate the performances of the RUO FAST™ System and the FAST-PBC Prep™ cartridges-generated LC for MALDI-TOF MS ID, manual and automated AST and rapid resistance detection testing. The study also evaluated the advantages and drawbacks of this approach in comparison with other conventional and rapid techniques currently available for the routine laboratory management of PBCs.

## Materials and methods

The study was conducted at the microbiology laboratory of the Cliniques universitaires Saint-Luc – UCL, a 960-bed tertiary hospital in Brussels, Belgium. Blood specimens from patients with a suspected bloodstream infection were inoculated into blood culture bottles (BD Bactec™ Plus Aerobic, Peds Plus and Lytic Anaerobic media, Becton Dickinson, Franklin Lanes, NJ, USA) and incubated 24 h a day, 7 days a week in BD Bactec™ FX devices (BD Diagnostic Systems, Sparks, MD, USA) for a standard 5-day period. SOC management of PBCs was performed during laboratory working hours (7 AM–0 AM, 7 days per week) and detailed as the “modified workflow” in a previous publication (Verroken et al., 2016). Downstream testing was performed either on an early subculture for blood cultures detected positive between 0 and 10 AM, either directly on the PBC for blood cultures detected positive between 10 AM and 3 PM, either on an overnight subculture for blood cultures detected positive between 3 PM and 0 AM. The MALDI Biotyper^®^ smart system (Bruker Daltonik GmbH, Bremen, Germany) was used for MALDI-TOF MS ID. Rapid resistance detection tests included the Clearview™ PBP2a SA Culture Colony Test (PBP2a; Abbott Diagnostics, Scarborough, ME, USA) performed on all *Staphylococcus aureus* and the βLACTA™ Test (βLT; Bio-Rad, Marnes-la-Coquette, France) performed on all Enterobacterales (EB) excluding chromosomal AmpC producers. AST for staphylococci, enterococci and EB was performed using the BD Phoenix System™ (Becton Dickinson, Franklin Lakes, NJ, USA) and AST of other bacteria (streptococci and *Pseudomonas aeruginosa*) was performed by disk diffusion using filter paper disks (Bio-Rad, Marnes-la-Coquette, France). All AST results were interpretated according to the breakpoint tables for interpretation of MICs and zone diameters of The European Committee on Antimicrobial Susceptibility Testing (EUCAST) version 10.0 (valid from 2020.01.01) (The European Committee on Antimicrobial Susceptibility Testing, 2020). Detection of extended-spectrum-β-lactamases (ESBL) and derepressed AmpC β-lactamases in EB relied on combination disk testing (ESBL + AmpC Screen Kit, Rosco diagnostic, Taastrup, Denmark). Carbapenemase resistance was characterized with immunochromatographic testing using the Resist-5 OOKNV and IMP K-SeT (Coris, BioConcept, Gembloux, Belgium) enabling the detection of the OXA-163, OXA-48, KPC, NDM, VIM and IMP genes.

### Study design

The study was conducted in two arms. In the initial prospective arm going over a 3-month period, FAST™ System testing was performed on the first positive-detected blood culture bottle of each patient with a monomicrobial Gram-stain result. In the second arm of the study, FAST™ System testing was performed on blood culture bottles spiked with multidrug-resistant bacteria selected from a patient strain bank stored at minus 20°C. Microorganisms and resistance profiles selected for the second arm are detailed in Table 1. Following three successive subcultures of the initially frozen strain, the spiking process consisted of inoculating the bottles (BD Bactec™ Plus Aerobic, Peds Plus and Lytic Anaerobic) with 10 ml of human blood from healthy volunteers and 10 μl of a 1,000-times dilution of a 0.5McF suspension from a fresh overnight subcultured isolate. Blood culture bottles were incubated in a BD Bactec™ FX device until they flagged positive.

**TABLE 1:** Microorganisms and resistance profiles selected for FAST™ System testing evaluation in the second arm of the study.

Gram-positive bacteria	*n*	Oxacilline R			
Staphylococci					
*Staphylococcus aureus*	15	8			
*Staphylococcus epidermidis*	7	4			
*Staphylococcus haemolyticus*	2	2			
*Staphylococcus hominis*	1	1			

**Gram-negative bacteria**	***n***	**Derepressed AmpC**	**ESBL**	**Carbapenemase**	**Chromosomic carbapenem R**

Enterobacterales					
*Citrobacter freundii*	1	1	0	0	0
*Enterobacter cloacae* complex	5	0	2	2 NDM, 1 OXA-48	0
*Escherichia coli*	13	2	10	1 NDM	0
*Enterobacter aerogenes*	2	2	0	0	0
*Klebsiella pneumoniae*	9	0	7	0	1
*Proteus mirabilis*	2	0	1	0	0
*Proteus vulgaris*	1	0	0	0	0
Non fermenters					
*Pseudomonas aeruginosa*	22	0	0	5 VIM	4

ESBL, extended-spectrum beta-lactamase; R, resistance.

### FAST™ System

**FIGURE 1:**
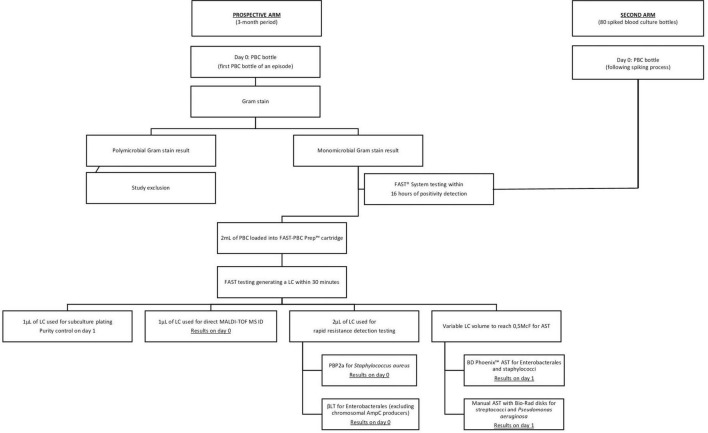
FAST™ System testing flowchart. AST, antimicrobial susceptibility testing; ID, identification; LC, liquid colony; PBC, positive blood culture; PBP2a, Clearview™ PBP2a SA Culture Colony Test; βLT, βLACTA Test.

The FAST™ System testing flowchart is presented in Figure 1. Following the availability of a monomicrobial Gram-stain result, 2 ml of the PBC was sampled into a FAST-PBC Prep™ cartridge which was loaded into the FAST™ System. Upon a 30-min automated lysis/centrifugation process, a LC constituted of pure viable bacteria was obtained. According to the manufacturer’s requirements, processing had to be performed within 16 h of blood culture positivity. Following the recovery of the LC, 1 μl was plated on a non-selective blood agar in order to verify purity on the next day. Then 1 μl was double-spotted with 1 μl of formic acid and 1 μl of matrix on a target for MALDI-TOF MS ID. Depending on the identified strain, rapid resistance detection testing was subsequently performed using 2 μl of the LC and following manufacturers’ recommendations. Ultimately AST was performed requiring a variable LC volume to obtain a standardized 0.5McF suspension. Rapid resistance detection tests and AST approaches were identically applied as in SOC management. In the prospective arm, AST from LC was exclusively performed if also done through SOC workflow. AST performances of FAST™ System testing were exclusively evaluated on staphylococci, enterococci, streptococci, EB and *P. aeruginosa*. AST on other microorganisms were not assessed in this study as the number of positive samples was too small to produce valuable data.

### Performance evaluation

All results obtained following FAST™ System testing were compared to SOC results considered as the reference. ID and AST discordances were verified through repeated testing from LC subculture and SOC overnight subculture. Discordant PBP2a and βLT results were evaluated upon the following day with AST results.

### Identification

MALDI-TOF MS ID results from the LC were interpreted according to a defined cut-off score of 1.7 for acceptable ID to the species level. A score under cut-off led to a single repetition of LC spotting and MALDI-TOF MS testing within the same day.

### Rapid resistance detection testing

Rapid resistance detection testing performances were evaluated by calculating sensitivity and specificity.

### Antimicrobial susceptibility testing

AST comparison was performed in accordance with Cumitech 31A recommendations for the verification and validation of procedures in the clinical microbiology laboratory (Clark et al., 2009). AST results comparison between FAST™ System testing and SOC was expressed in categorical agreement (CA) percentage (total categorical matches/total antibiotics tested × 100). Discordances were classified into very major errors (VME: false susceptibility with AST performed on LC), major errors (ME: false resistance with AST performed on LC) and minor errors (MinE: reference test result susceptible at increased exposure or in the area of technical uncertainty while AST performed on LC susceptible or resistant, or vice versa). The VME rate was calculated by dividing the number of VME by the number of resistant bacteria (reference method) × 100 and The ME rate was calculated by dividing the number of ME by the number of susceptible bacteria (reference method) × 100. MinE rate was calculated by dividing the number of MinE by the total number of strains tested × 100. Acceptable performance rates for CA should be ≥90%, whereas acceptable performance for the VME rate should be ≤3%. The ME rate should be ≤3%. For ME and MinE combined, the error rate should be combined ≤7%.

## Results

In the prospective arm, FAST™ System testing was performed on 198 patient PBCs. Ten samples were excluded from analysis because they were polymicrobial on the purity control plate on day one, 1 sample was discarded due to an instrument error and 1 sample did not have a final SOC ID. In the second arm of the study FAST™ System testing was performed on 80 spiked PBC.

### Identification results

**FIGURE 2:**
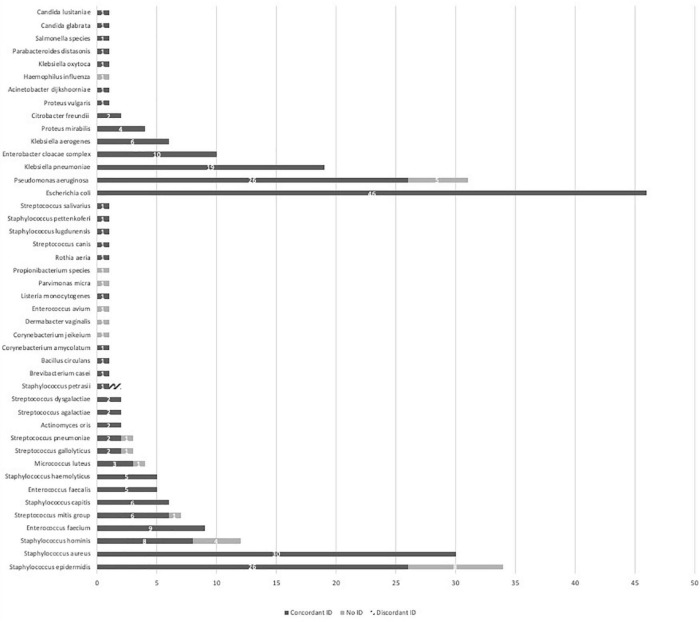
MALDI-TOF MS identification performances from a liquid colony following FAST™ System testing on positive blood cultures in both study arms. ID, identification; MALDI-TOF MS, matrix-assisted laser desorption ionization time-of-flight mass spectrometry.

Complete data are presented in Figure 2. Overall concordant ID was observed in 238/266 (89.5%) PBC with a mean MALDI-TOF MS score from LC testing of 2.1. Gram-positive bacteria, Gram-negative bacteria and yeast reached concordant ID results of respectively, 118/140 (84.3%), 118/124 (95.2%) and 2/2 (100%). An insufficient score resulting in the absence of ID was observed in 27/266 (10.2%) PBC. Among the non-identified PBC, 21 concerned Gram-positive bacteria including 8 *Staphylococcus epidermidis*, 4 *Staphylococcus hominis*, 3 streptococci, 1 enterococcus and 5 other strains most of the time considered as blood culture bottle contaminants. Six concerned Gram-negative bacteria including 5 *P. aeruginosa* and 1 *Haemophilus influenza*. Ultimately 1/266 (0.4%) PBC ID led to a discordant result. A *Staphylococcus petrasii* was erroneously identified as *Staphylococcus capitis* with MALDI-TOF MS from the LC with an ID score of 1.93.

### Rapid resistance detection test results

PBP2a was evaluated on a total of 30 *S. aureus* including 10 methicillin-resistant *S. aureus*. Sensitivity and specificity of PBP2a testing from a LC compared to SOC PBP2a were, respectively, 100% (10/10) and 75% (15/20). βLT was performed on 66 EB including 22 ESBL strains, one NDM-producing *Escherichia coli* and one ESBL *Klebsiella pneumoniae* with a chromosomic carbapenem resistance. Sensitivity and specificity of βLT testing from a LC compared to SOC βLT were, respectively, 95.8% (23/24) and 100% (42/42). A false negative LC βLT result on the NDM-producing *E. coli* was observed.

### Antimicrobial susceptibility testing results

Automated AST was performed through both SOC testing and from a LC on a total of 127 PBC counting 54 Gram-positive strains and 73 EB. In addition, disk diffusion AST was compared between SOC testing and from a LC on 8 streptococci and 25 *P. aeruginosa*. Importantly, AST testing from LC could not be performed on 13/173 (7.5%) PBC pathogens due to insufficient LC biomass to obtain a 0.5 McF concentration.

Table 2 illustrates result comparison of automated AST on staphylococci and enterococci including 26 *S. aureus*, 15 S. coagulase negative, 8 *Enterococcus faecium* and 5 *E. faecalis*. CA, VME, ME and MinE rates were, respectively, 97.7% (650/665), 1.4% (2/144), 1.9% (9/486) and 1.3% (4/300). Moxifloxacin and tobramycin were false susceptible using the LC for 2 distinct *S. aureus* isolates. The majority of ME were found with Tobramycin 5/28 (17.9%).

**TABLE 2:** Results from automated AST (BD Phoenix™ System) of 54 staphylococci and enterococci from a liquid colony following FAST™ System testing on positive blood cultures in both study arms combined.

Staphylococci and enterococci *n* = 54				
Antibiotic	CA (%)	VME (%)	ME (%)	MinE (%)
Amikacin	40/41 (97.6)	0/7 (0)	1/34 (2.9)	0/41 (0)
Ampicillin	13/13 (100)	0/8 (0)	0/5 (0)	0/13 (0)
Cefoxitin	26/26 (100)	0/9 (0)	0/17 (0)	NA
Ciprofloxacin	41/41 (100)	0/12 (0)	0/0	0/41 (0)
Clindamycin	41/41 (100)	0/12 (0)	0/29 (0)	0/41 (0)
Erythromycin	41/41 (100)	0/21 (0)	0/20 (0)	0/41 (0)
Gentamicin	39/41 (95.1)	0/8 (0)	2/33 (6.1)	NA
Gentamicin (HLR)	13/13 (100)	0/4 (0)	0/9 (0)	NA
Linezolid	54/54 (100)	0/0	0/54 (0)	NA
Moxifloxacin	40/41 (97.6)	1/12 (8.3)	0/29 (0)	NA
Oxacillin	41/41 (100)	0/18 (0)	0/23 (0)	NA
Rifampicin	41/41 (100)	0/3 (0)	0/38 (0)	0/41 (0)
Teicoplanin	54/54 (100)	0/0	0/54 (0)	NA
Tetracycline	38/41 (92.7)	0/6 (0)	0/30 (0)	3/41 (7.3)
Trimethoprim-sulfamethoxazole	39/41 (95.1)	0/11 (0)	1/29 (3.4)	1/41 (2.4)
Tobramycin	35/41 (85.4)	1/13 (8.0)	5/28 (17.9)	NA
Vancomycin	54/54 (100)	0/0	0/54 (0)	NA
**Total**	650/665 (97.7)	2/144 (1.4)	9/486 (1.9)	4/300 (1.3)

CA, categorical agreement; VME, very major error; ME, major error; MinE, minor error; NA, not applicable.

Table 3 shows data comparison of automated AST on 73 EB including 42 *E. coli*, 13 *K. pneumoniae*, 6 *Enterobacter cloacae* complex, 6 *Klebsiella aerogenes*, 2 *Citrobacter freundii*, 2 *Proteus mirabilis*, 1 *Klebsiella oxytoca* and 1 *Proteus vulgaris*. CA, VME, ME and MinE rates were 97.8% (1311/1340), 1.4% (6/427), 0.1% (1/771) and 2.8% (22/788) respectively. VME were observed in 6 distinct EB strains and 5 different antibiotics. The single ME was seen with gentamicin and *P. vulgaris*.

**TABLE 3:** Results from automated AST (BD Phoenix™ System) of 73 Enterobacterales from a liquid colony following FAST™ System testing on positive blood cultures in both study arms combined.

Enterobacterales *n* = 73				
Antibiotic	CA (%)	VME (%)	ME (%)	MinE (%)
Amikacin	73/73 (100)	0/7 (0)	0/66 (0)	NA
Amoxicillin-clavulanic acid	72/73 (98.6)	1/43 (2.3)	0/30 (0)	NA
Ampicillin	73/73 (100)	0/59 (0)	0/14 (0)	NA
Cefepime	70/73 (95.9)	0/24 (0)	0/43	3/73 (4.1)
Ceftriaxone	69/73 (94.5)	2/35 (5.7)	0/37 (0)	2/73 (2.7)
Ceftazidime	73/73 (100)	0/28 (0)	0/40 (0)	0/73 (0)
Cefuroxime iv	58/58 (100)	0/27 (0)	0/0	0/58 (0)
Ciprofloxacin	67/73 (91.8)	0/29 (0)	0/37	6/73 (8.2)
Ertapenem	73/73 (100)	0/10 (0)	0/63 (0)	NA
Gentamicin	71/73 (97.3)	1/14 (7.1)	1/59 (1.7)	NA
Imipenem	71/73 (97.3)	0/3 (0)	0/66 (0)	2/73 (2.7)
Levofloxacin	71/73 (97.3)	0/28 (0)	0/39 (0)	2/73 (2.7)
Meropenem	73/73 (100)	0/3 (0)	0/67 (0)	0/73 (0)
Piperacillin	72/73 (98.6)	0/50 (0)	0/22 (0)	1/73 (1.4)
Piperacillin-tazobactam	67/73 (91.8)	1/19 (5.3)	0/50 (0)	5/73 (6.8)
Tigecycline	41/41 (100)	0/0	0/41 (0)	NA
Trimethoprim-sulfamethoxazole	71/73 (97.3)	1/30 (3.3)	0/42 (0)	1/73 (1.4)
Tobramycin	73/73 (100)	0/18 (0)	0/55 (0)	NA
**Total**	1311/1340 (97.8)	6/427 (1.4)	1/771 (0.1)	22/788 (2.8)

CA, categorical agreement; VME, very major error; ME, major error; MinE, minor error; NA, not applicable.

Disk diffusion AST performances using the LC colony were evaluated on 8 streptococci including 3 *Streptococcus agalactiae*, 2 *Streptococcus dysgalactiae* and 3 *Streptococcus mitis* group. CA reached 100% with a total of 41 antibiotic combinations tested as presented in Table 4.

**TABLE 4:** Results from disk difffusion AST (filter paper disks, Bio-Rad) of 8 streptococci from a liquid colony following FAST™ System testing on positive blood cultures in both study arms combined.

Streptococci *n* = 8				
Antibiotic	CA (%)	VME (%)	ME (%)	MinE (%)
Benzylpenicillin	8/8 (100)	0/2 (0)	0/6 (0)	0/8 (0)
Clindamycin	8/8 (100)	0/2 (0)	0/6 (0)	0/8 (0)
Erythromycin	5/5 (100)	0/2 (0)	0/3 (0)	0/5 (0)
Minocycline	5/5 (100)	0/3 (0)	0/2 (0)	0/5 (0)
Moxifloxacin	5/5 (100)	0/1 (0)	0/4 (0)	NA
Rifampicin	5/5 (100)	0/0	0/5 (0)	0/5 (0)
Trimethoprim-sulfamethoxazole	5/5 (100)	0/0	0/5 (0)	0/5 (0)
**Total**	41/41 (100)	0/10 (0)	0/31 (0)	0/36 (0)

CA, categorical agreement; VME, very major error; ME, major error; MinE, minor error; NA, not applicable.

Ultimately AST was assessed on 25 *P. aeruginosa* resulting in a CA of 99% (297/300), no VME, no ME and 3/250 (1.2%) MinE as detailed in Table 5. Altogether AST using the LC resulted in acceptable rates according to Cumitech criteria for all evaluated automated and manual AST approaches.

**TABLE 5:** Results from disk difffusion AST (filter paper disks, Bio-Rad) of 25 *Pseudomonas aeruginosa* from a liquid colony following FAST™ System testing on positive blood cultures in both study arms combined.

*P. aeruginos*a *n* = 25				
Antibiotic	CA (%)	VME (%)	ME (%)	MinE (%)
Amikacin	25/25 (100)	0/4 (0)	0/21 (0)	NA
Aztreonam	23/25 (92)	0/2 (0)	0/0	2/25 (8)
Cefepime	24/25 (96)	0/6 (0)	0/0	1/25 (4)
Ceftazidime	25/25 (100)	0/7 (0)	0/0	0/25 (0)
Ciprofloxacin	25/25 (100)	0/7 (0)	0/0	0/25 (0)
Imipenem	25/25 (100)	0/8 (0)	0/0	0/25 (0)
Meropenem	25/25 (100)	0/7 (0)	0/17 (0)	0/25 (0)
Piperacillin	25/25 (100)	0/8 (0)	0/0	0/25 (0)
Piperacillin-tazobactam	25/25 (100)	0/8 (0)	0/0	0/25 (0)
Ticarcillin	25/25 (100)	0/7 (0)	0/0	0/25 (0)
Ticarcillin-clavulanic avid	25/25 (100)	0/8 (0)	0/0	0/25 (0)
Tobramycin	25/25 (100)	0/4 (0)	0/21 (0)	NA
**Total**	297/300 (99)	0/76 (0)	0/59 (0)	3/250 (1.2)

CA, categorical agreement; VME, very major error; ME, major error; MinE, minor error; NA, not applicable.

## Discussion

For many years, numerous laboratories have developed their own manual, in-house techniques to concentrate microorganisms from PBCs aiming to perform direct downstream testing. Throughout time, automated AST systems were evaluated for their combined performances of ID and AST directly from a PBC. In 2012, Gherardi et al. performed a comparative evaluation of the Vitek™ (bioMérieux, Marcy l’Etoile, France) and BD Phoenix™ systems for rapid ID and AST, from a standardized bacterial pellet obtained through various centrifugation steps of the PBC (Gherardi et al., 2012). Altogether, 100 and 92.3% of the Gram-negative isolates and 75 and 43.75% of the Gram-positive isolates showed concordant ID between the direct and standard methods with Vitek™ and BD Phoenix™, respectively. Additionally, AST CA of 98.7 and 99% in Gram-negative and of 96.2 and 99.5% in Gram-positive isolates with Vitek™ and BD Phoenix™, respectively, were observed. Historically multiple laboratories have performed similar evaluations yet with reduced hands-on time of pellet preparation steps. Reported performances of direct MALDI-TOF MS ID for Gram-negative bacteria exceeded 95%. However, a much lower SOC concordance rate of 79% was reached for Gram-positive bacteria (Maelegheer and Nulens, 2017; Hogan et al., 2019; Infante et al., 2021). In addition, these studies reported a CA that varied according to the evaluated AST automate between 92.9 and 98.9% using the PBC-derived pellet. FAST™ System testing results from our study were at least equal or surpassed the latter with an ID SOC concordance reaching 84.3 and 95.2% for Gram-positive and Gram-negative bacteria, respectively. Considering Gram-positive bacteria, failure of ID principally concerned strains that are often considered as contaminants in PBCs. The *S. capitis* erroneously identified as *S. petrasii* was a contaminant and repeated testing from LC subculture ultimately identified a *S. capitis.* It could be supposed that the PBC was most probably a polymicrobial blood culture. The absence of ID among Gram-negative bacteria principally concerned *P. aeruginosa* strains all originating from the spiked blood culture bottles from the second arm of the study. The conservation and spiking process might have altered the quality of the strains and hereby reduced the MALDI-TOF MS ID performances from LC. No other bacteria showed similar lower ID performances in the second arm of the study versus the prospective arm. AST analysis involving disk diffusion and BD Phoenix™ testing, showed excellent performances substantially outperforming Cumitech requirements for all groups of bacteria frequently identified in PBC (Clark et al., 2009). Completing the prospective arm of the study with FAST™ System testing on spiked blood cultures aimed to broaden AST evaluation on multidrug-resistant bacteria and did not increase VME rates. The sporadic VME involved different antibiotics tested on distinct bacteria allowing us to conclude that no specific trend was present. Nine of the 10 ME in our study were with aminoglycosides, however, their limited use as a targeted treatment of bacteriemia downplays the clinical impact of this observation. Performance data on the FAST™ System generated LC are very scarce as the approach has only been marketed very recently. Similar analysis using the FAST™ System testing-generated LC reported MALDI-TOF MS ID concordances of 94% and CA with Vitek™ 2 for Gram-positive and Gram-negative bacteria of 97.4 and 98.5%, respectively (Grinberg et al., 2022).

An added value of this study was the evaluation of rapid resistance detection testing performed on the LC. PBP2a testing using the LC showed suboptimal results as 5/20 methicillin-susceptible *S. aureus* (MSSA) PBC were erroneously tested positive. Initially observed in the prospective arm with 3 false positive PBP2a results on a total of 14 MSSA strains, the issue was confirmed in the second arm of the study with 2 false positive PBP2a results among 7 MSSA. All PBP2a testing results in the SOC workflow were in concordance with AST results. To our knowledge, similar observations were not reported by others. Satisfying sensitivity and specificity performances were reported by Kolesnik-Goldmann et al. who evaluated PBP2A testing on 4–6 h *S. aureus* subcultures (Kolesnik-Goldmann et al., 2021). Similarly, Defourny et al. reported a 100% sensitivity and specificity of PBP2A directly performed on a home-made PBC pellet (Defourny et al., 2014). A likely hypothesis that could explain the poor specificity results of this study is a cross-reaction of one of the reagents included in the FAST-PBC Prep™ cartridge with the recombinant monoclonal antibody fragments of the test membrane. While awaiting the conclusions of additional analyses currently performed at Qvella’s, extreme vigilance is recommended in the interpretation of negative PBP2a results obtained from a LC. On the contrary, βLT study results showed very satisfying performances and hereby confirmed previous results on EB excluding AmpC chromosomal producers (Prod’hom et al., 2015).

Despite a monomicrobial Gram stain, 10 PBC of the prospective arm were ultimately excluded from data analysis as they led to a polymicrobial growth on the subcultured purity control plate on day 1. Nevertheless, when used in routine FAST™ system test results would have already been made available prior to the polymicrobial detection. Considering the 10 PBC, 3 PBC tested with the FAST™ system did not lead to any ID results and 7 PBC enabled ID of 1 out of the 2 strains. There were no erroneous IDs. The lack of ability of MALDI-TOF MS to detect all micro-organisms in mixed cultures through direct ID is well-known and reported in various publications (Verroken et al., 2015). Culturing a purity plate remains therefore essential.

While awaiting the availability of innovative sepsis diagnostic tools skipping blood incubation, a plethora of methods to speed up results from PBCs are existing (Dubourg et al., 2018; Peker et al., 2018). The FAST™ System using a LC belongs to the category of techniques aiming at the rapid production of a “microorganism pellet or suspension” with the same characteristics as an overnight subcultured colony allowing immediate downstream testing. This distinct approach combines several advantages. First of all, ID can be performed with MALDI-TOF MS enabling access to a nearly “universal” bank of bacterial fingerprints in contrast with direct molecular methods giving access to a restricted panel of strain IDs with previous publications reporting 85.2–89.1% coverage of PBC organisms (Verroken et al., 2019; Ullberg and Özneci, 2020). Additionally, no sacrifices have to be made on the selection of tested antibiotics for AST. Recent commercial AST tools designed for direct testing on PBC use a restricted number of antibiotics and don’t allow the selection of a panel in accordance with the local resistance strain epidemiology of each medical center (Charnot-Katsikas et al., 2017; Boland et al., 2019; Grohs et al., 2021). FAST™ System testing enables the use of SOC well-known largely validated antimicrobial approaches including disk diffusion or automated AST, initiated from a standardized inoculum hereby enhancing the accuracy of the AST analytical performances. A procedural benefit of the FAST™ System is the complete automation of the approach with human intervention being limited to the sampling of 2 ml PBC into a cartridge and minimal run start time. Maximum number of samples that can be run at once are 2 PBC tests. The short 30-min turn-around-time for the creation of the LC enables a testing flow of approximately 20 PBC/day knowing that only the first PBC of an episode ultimately requires speeded-up testing. In a clinical microbiology laboratory that does not have a night shift, FAST™ System testing could be performed on blood cultures detected positive until approximately 5 PM ensuring ID on the same day and AST results the following day. Eventually the use of a commercial approach for SOC microbiology testing facilitates the process toward accreditation as only a method verification is required in contrast to usually fastidious and broad method validations for in-house approaches.

Originally the design of our study was not thought to integrate time-to-result measurements compared to SOC. FAST™ System testing was performed throughout the workday right upon PBC detection yet depending on the availability of the device. Subsequently, downstream testing was done in batches in the late morning and late afternoon. We can therefore affirm that ID and rapid resistance detection test results were available on the day the blood culture flagged positive within a maximum time period of 5 h following FAST™ System testing while the first AST results were available in the late evening or during the night. These turn-around-times are aligned and even beyond targeted SOC timeframe objectives of PBC management in clinical microbiology laboratories. How the FAST™ System can be integrated in the SOC PBC workflow to optimize its time-saving advantage will definitely vary from one laboratory to another depending on laboratories’ working hours, currently-used downstream tests but also the existing interactions with clinicians as well as ongoing antimicrobial stewardship programs. Important to note is the recent overview presented by Lamy et al. illustrating that progress in PBC management should be based on a bundle approach joining rapid diagnostics with pre-analytical improvements, optimized microbiologistics and customized result communication (Lamy et al., 2020).

Eventually a new technology is only fully effective upon the demonstration of its clinical impact. In a recent review, Banerjee et al. concluded that rapid AST methods on PBC can shorten time to optimal treatment and improve antibiotic stewardship, however, they did not demonstrate significant reductions in mortality, length of stay or adverse effects (Banerjee and Humphries, 2021). It is therefore essential to set up well-designed clinical randomized controlled trials targeting specific patient populations and promoting clinicians’ interactions to value the real impact of FAST™ System testing.

In conclusion, our results show that generating a LC through FAST™ System testing is a reliable approach to speed up downstream testing of a PBC with satisfying performances considering MALDI-TOF MS ID, disk diffusion and BD Phoenix™ AST. Next steps include its optimal integration into SOC PBC routine workflow and the set-up of effective clinical and economical studies.

## Data availability statement

The original contributions presented in this study are included in the article/supplementary material, further inquiries can be directed to the corresponding author.

## Ethics statement

The studies involving human participants were reviewed and approved by Comité d’Éthique Hospitalo-Facultaire, Cliniques Universitaires Saint-Luc. Written informed consent for participation was not required for this study in accordance with the national legislation and the institutional requirements.

## Author contributions

AV designed the study and analyzed all the results and wrote the manuscript. CH, FB, and JC performed the experiences. AA and HR-V provided critical feedback and contributed to the final version of the manuscript. All authors approved the submitted version.
